# Impact of statin intake on malignant hyperthermia: an in vitro and in vivo swine study

**DOI:** 10.1186/s12871-020-01186-5

**Published:** 2020-10-23

**Authors:** Asensio Gonzalez, Tinen L. Iles, Paul A. Iaizzo, Oliver Bandschapp

**Affiliations:** 1 Department for Anesthesia, Interdisciplinary Intermediate Care, Prehospital Emergency Medicine and Pain Therapy, University Hospital Basel, Spitalstrasse 21, CH-4031 Basel, Switzerland; 2 Department of Surgery and Integrative Biology and Physiology, Institute for Engineering in Medicine, University of Minnesota, Minneapolis, USA

**Keywords:** Malignant hyperthermia, Muscle disease, Statin medication, MHS swine

## Abstract

**Background:**

Statin intake is associated with muscular side effects, among which the unmasking of latent myopathies and of malignant hyperthermia (MH) susceptibility have been reported. These findings, together with experimental data in small animals, prompt speculation that statin therapy may compromise the performance of skeletal muscle during diagnostic in vitro contracture tests (IVCT). In addition, statins might reduce triggering thresholds in susceptible individuals (MHS), or exacerbate MH progression. We sought to obtain empirical data to address these questions.

**Methods:**

We compared the responses of 3 different muscles from untreated or simvastatin treated MHS and non-susceptible (MHN) pigs. MHS animals were also invasively monitored for signs of impending MH during sevoflurane anesthesia.

**Results:**

Muscles from statin treated MHS pigs responded with enhanced in vitro contractures to halothane, while responses to caffeine were unaltered by the treatment. Neither agent elicited contractures in muscles from statin treated MHN pigs. In vivo, end- tide pCO2, hemodynamic evolution, plasma pH, potassium and lactate concentrations consistently pointed to mild acceleration of MH development in statin-treated pigs, whereas masseter spasm and rigor faded compared to untreated MHS animals.

**Conclusions:**

The diagnostic sensitivity and specificity of the IVCT remains unchanged by a short-term simvastatin treatment in MHS swine. Evidence of modest enhancement in cardiovascular and metabolic signs of MH, as well as masked pathognomonic muscle rigor observed under simvastatin therapy suggest a potentially misleading influence on the clinical presentation of MH. The findings deserve further study to include other statins and therapeutic regimes.

## Background

Statins are widely prescribed drugs to treat hypercholesterolemia due to a safe profile and significant efficacy at reducing cardiovascular-related morbidities in coronary artery disease patients, as well as in healthy individuals [1, 2]. A growing number of daily statin users worldwide report associated muscular side effects, ranging from mild myalgia or exertional fatigue to severe rhabdomyolysis [3, 4]. Latent metabolic [5, 6], inflammatory or autoimmune myopathies [7–9] have also been disclosed after statin treatment. These may be preexisting subclinical pathologies of the skeletal muscle that often remain apparent even after statin withdrawal [6]. Some of them reflect associated genetic predispositions [10].

Physiological studies of the isolated ryanodine receptor RyR1 in lipid bilayers recently characterized an interaction with simvastatin which suggested that this drug may facilitate calcium ion leakage [11], a known feature of MHS muscle. Acute exposure to statins in vitro elicits contractures in muscle from MH-susceptible (MHS) pigs but not in muscle from non-susceptible (MHN) animals [12]. Experimental studies have shown that simvastatin administration in vivo precipitates a hypermetabolic crisis resembling MH in transgenic mice with targeted pathogenic *RyR1* gene mutations [13]. Moreover, malignant hyperthermia susceptibility has been observed following statin treatment [14, 15], an association that has received support from inferential epidemiological data [16].

The clinical, epidemiological and experimental findings combined suggest that muscle function is compromised under statin treatment, which could interfere with the diagnostic screening of suspect MHS probands by the in vitro contracture test (IVCT). Moreover, it is unknown whether the onset or the progression of MH episodes in vivo is negatively affected by statins in MHS individuals. We obtained direct insight on these subjects by evaluating the diagnostic efficacy of the IVCT in muscles from MHS pigs treated with a short-term simvastatin regime. We also investigated whether statin therapy may in itself induce false positive IVCT results in muscles from non-susceptible pigs. Finally, we monitored the progression of cardiovascular and metabolic variables during MH episodes triggered by sevoflurane anesthesia in treated and untreated animals.

## Methods

### Animal model

The study and the experimental protocol were approved and conducted in accordance to the Institutional Animal Care and Use Committee of the University of Minnesota (IACUC, ID 1308-30893A, Minneapolis, USA). Six MHS Pietrain pigs (Boyle farms, Moorehead, IA, USA), 6 months old and all from the same litter were studied, 4 treated daily with 40 mg simvastatin p.o. for 4 weeks and 2 untreated as MHS controls. Five additional Yorkshire pigs (Manthei hog farm, Elk river, MN, USA), 2 under the same simvastatin regime and 3 untreated, underwent identical procedures and were used to evaluate the specificity of the in vitro contracture test. During all the experiments and when assessing the results, the study team was blinded and not aware of the treatment group the animals were in. The order in which the animals were tested was randomly assigned by a researcher who was not involved in the actual study.

### In vitro contracture tests, specimen viability and muscle excitability

In vitro experiments were performed in 3 skeletal muscles with different fiber composition: white *vastus lateralis* (composed mostly of fast, type II fibers), *rectus abdominis* (mixed fiber type), and diaphragm (mixed, mostly type I fibers). Muscle pieces from ventilated, living animals were excised for IVCT prior to exposure of the pigs to sevoflurane (detailed in the next section), immediately transported to the lab, and dissected under carbogenated Krebs buffer at room temperature. The specimens were tied with silk sutures to form 30–40 mm long bundles, suspended in 40 ml chambers filled with Krebs solution under 2 g of tension, and stimulated with electrical field pulses of 1 ms duration and supramaximal voltage at 0.1 Hz. An equilibration period of 30–45 min preceded each IVCT, and specimens exhibiting twitch peak amplitudes below 1 g were systematically discarded (Table 1). Tension was recorded with isometric Grass F07 force transducers interfaced to a digital acquisition system at a 1000 Hz sampling rate. Viable muscle bundles were exposed to cumulative doses of halothane (0.5 to 3%) or caffeine (0.5 to 32 mmol. L^− 1^) at 3-min intervals, following the protocol of the European MH Group [17]. Tension data (in g) were then normalized by cross-sectional area (in cm^2^), calculated as CSA = W/(L*1.056), where bundle weight (W) and length (L) were measured with a caliper at the end of each experiment, and 1.056 g/cm^3^ represents the average density of skeletal muscle.

**Table 1:** Viability of muscle bundles prepared for in vitro studies

	MHS		MHN	
	Untreated	Simvastatin	Untreated	Simvastatin
Number of bundles prepared^a^	122	203	156	64
Percentage of discarded bundles	23.8	32.5	6.4	26.6

^a^pooled from vastus, rectus and diaphragm

Additional muscle bundles were used for in vitro specimen viability and muscle excitability assessments. Specimens were considered non-viable when twitch contractions fell below 1 g during equilibration. Supramaximal stimulus threshold, the minimum voltage required to achieve maximal twitch contraction amplitude, was measured individually in 8 bundles of each muscle type per group (MHN-untreated, MHN-statin, MHS-untreated, and MHS-statin) (Table 2).

**Table 2:** Voltage thresholds for supramaximal twitch contraction in vitro

	MHS		MHN	
	Untreated	Simvastatin	Untreated	Simvastatin
Vastus	6.66 ± 1.1^†^	6.49 ± 1.6^‡^	11.9 ± 3.3	9.51 ± 2.4
Rectus	4.80 ± 1.6^†^	4.78 ± 1.2	10.2 ± 3.4	6.21 ± 2.5^§^
Diaphragm	7.76 ± 0.9	5.85 ± 1.6^§^	7.76 ± 1.1	6.73 ± 1.3

Mean ± S.D. *n* = 8 bundles per group.

^†^*p* ≤ 0.001 vs. MHN untreated, ^‡^*p* ≤ 0.001 vs. MHN simvastatin, ^§^*p* ≤ 0.01 vs. untreated

In each muscle type, normalized contractures from 4 specimens per trigger agent and per animal were pooled and compared between treated and untreated groups by the non-parametric Mann-Whitney test, as normally distributed data could not be assumed. Fisher’s exact test was used to compare viability in treated vs untreated MHS and MHN pigs by pooling all muscle samples from each treatment group. Voltage thresholds were averaged and compared by Mann-Whitney tests with significance set at *p* < 0.05. All statistical analyses were performed using Prism software package v8.4.3 (Graphpad Software, La Jolla, CA).

### In vivo monitoring of sevoflurane-induced MH episodes

Each animal was initially anesthetized with intramuscular Telazol (tiletamine HCl and zolazepam HCl; Fort Dodge Animal Health, Fort Dodge, IA), which was continued intravenously as required. After intubation, they were mechanically ventilated to achieve end-tidal pCO2 (etCO2) of 40 mmHg. A balloon-tipped catheter (Edwards Swan-Ganz Thermodilution Catheter, Irvine, CA) was inserted in the pulmonary artery to measure cardiac output and core temperature. Esophageal and rectal temperature were also monitored with additional thermal probes. Mean arterial pressure (MAP) was monitored through a femoral line. A specially designed pressure bulb [18] was inserted in the jaw to display pressure development by the masseter muscles and zeroed just prior to the administration of sevoflurane. Muscle specimens from *vastus, rectus* and diaphragm muscles were resected for IVCT, and sevoflurane was subsequently administered at an inspired concentration of 2.2%. Blood samples were drawn, initially every 10 min, and then every 5 min once MH-triggering was noticed, until study endpoints were reached. Endpoints were defined as asystole for MHS swine, or 90 min after sevoflurane administration started for MHN pigs, which were then euthanized via intravenous KCl. Thresholds for each variable were pre-defined as listed in Table 3. The average time (± SD) needed to reach each threshold in treated and untreated animals are reported.

**Table 3:** In vivo progression of simvastatin treated and untreated MHS pigs during sevoflurane anesthesia

Measurement	Threshold	Time (min) to reach threshold^**a**^	
		Statin (*N* = 4)	Untreated (*N* = 2)
End tidal pCO_2_	50 mmHg	19.6 (± 3.3)	22.2 (± 3.5)
Mean arterial pressure	50 mmHg	35.3 (± 7.9)	38 (± 1.4)
Core temperature	40 °C	47 (± 18.7)	52.5 (± 9.2)
Heart rate	Asystole	62.7 (± 11.2)	94.7 (± 44.5)
Blood PaCO_2_	50 mmHg	22.3 (± 4.8)	30.5 (± 7.8)
Blood pH	7.2	29.8 (± 6.3)	41 (± 7.1)
Blood K^+^	6 mmol. L^−1^	41 (± 7.1)	53.5 (± 10.6)
Blood lactate	10 mmol. L^− 1^	26 (± 7.1)	38.5 (± 10.6)

^a^Mean (± SD)

## Results

All MHS pigs were female and weighed 94.8 ± 3.7 kg in the treated group, and 93.3 ± 1.7 kg in the untreated group (mean ± SD). Statin treated MHN animals were females of 96.9 ± 4.1 kg. Untreated MHN pigs were males of 81.1 ± 3.8 kg.

### In vitro contracture tests

**Fig. 1:**
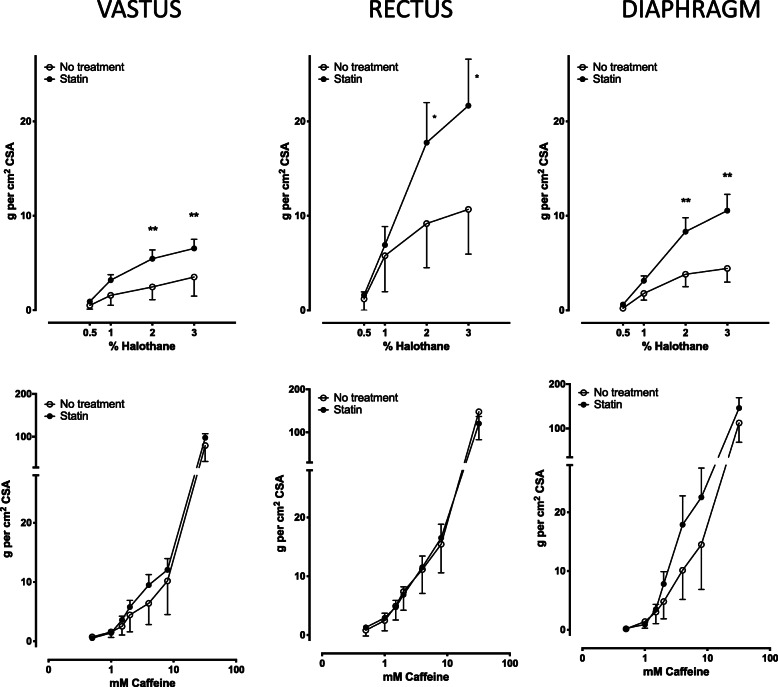
IVCT in 3 different muscles from MHS swine. Responses to halothane (upper panels) were increased in muscles from simvastatin-treated MHS pigs compared with untreated MHS pigs (*N* = 4 MHS statin treated animals, *N* = 2 MHS untreated animals; 4 bundles per muscle type per animal; * *p* < 0.05, ** *p* < 0.005)

Muscles from MHS pigs responded with contractures upon exposure to halothane or caffeine. The contractures elicited by halothane in *vastus, rectus*, and diaphragm muscles were larger in simvastatin treated than in untreated pigs (Fig. 1). Muscle responses to 2% halothane were significantly enhanced in treated vs. untreated animals and similar differences were observed at 3% halothane. By contrast, caffeine-induced contractures were similar in both treatment groups (Fig. 1).

Muscles from statin-pretreated MHN pigs did not respond to halothane (0.5–3%) or caffeine (0.5–4 mmol. L^− 1^) but did confirm specimen viability by responding with strong contractures to 32 mmol. L^− 1^ caffeine. Muscle bundles from untreated MHN pigs did not exhibit contractures in response to either agent but showed viability via contractures to 32 mmol. L^− 1^ caffeine.

### In vitro muscle viability and excitability

Muscle bundles from statin-treated (either MHS or MHN) pigs were often hypercontracted during dissection and showed unstable baselines and decaying twitch contractions during the equilibration period when compared to muscles from untreated pigs. Bundle replacement due to loss of viability during equilibration was significantly more frequent in statin-treated animals (*p* < 0.0001, *n* = 545, Table 1).

Statin treatment increased muscle excitability to electrical field stimulation in vitro in some muscle types, as reflected by lower supramaximal voltage thresholds (Table 2). In untreated MHS pigs, *vastus* and *rectus* muscles showed lower voltage thresholds than those from untreated MHN animals, indicating hyperexcitability of MHS muscles, but diaphragm thresholds were unchanged. Statin treatment in MHS animals did not affect the already low thresholds of *vastus* and *rectus*, but it did decrease diaphragm thresholds significantly. In MHN pigs, statin treatment significantly decreased voltage thresholds in *rectus* muscle only.

### In vivo monitoring of sevoflurane-induced MH

**Fig. 2:**
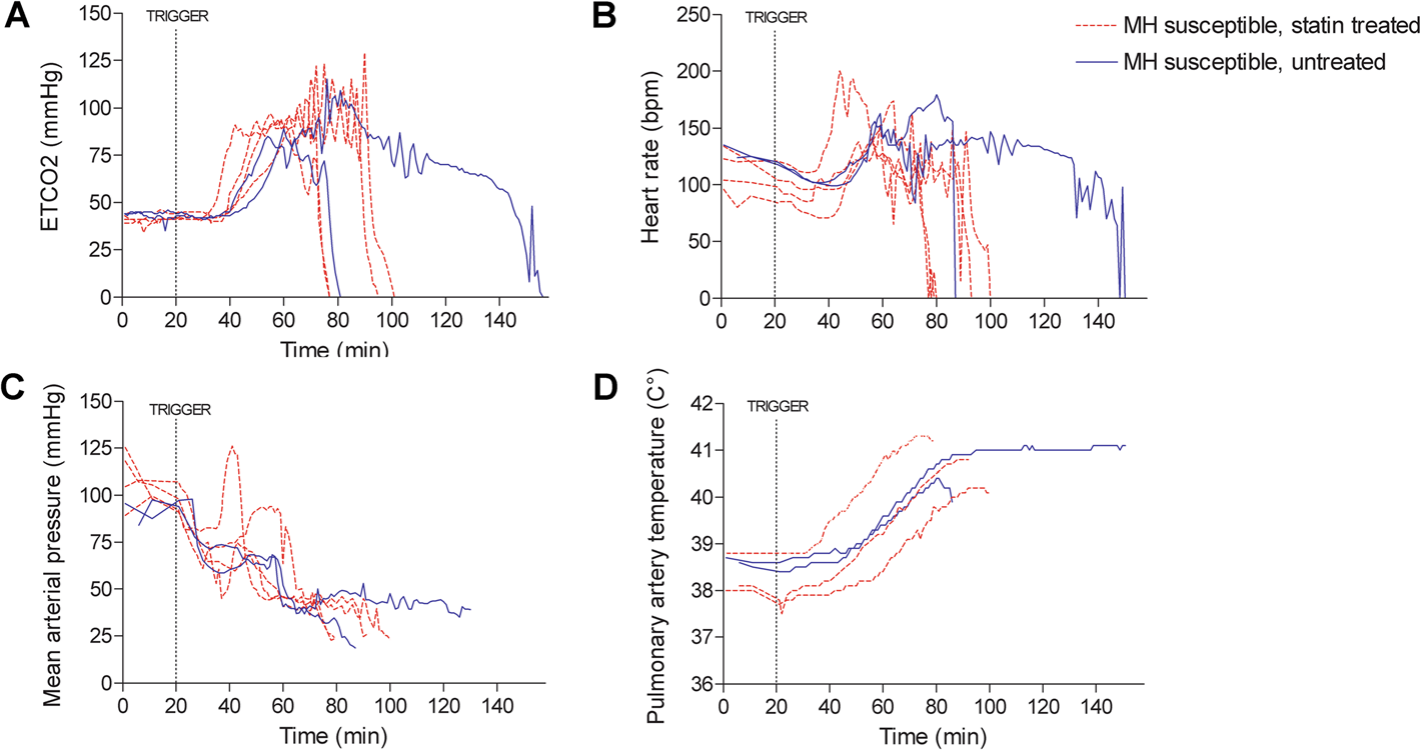
Cardiorespiratory, hemodynamic and thermal variables measured during sevoflurane-induced MH in susceptible swine treated with simvastatin (*N* = 4, discontinuous red line) and untreated susceptible swine (*N* = 2, blue line). The trigger line marks the start of sevoflurane administration at inspired concentrations of 2.2%

**Fig. 3:**
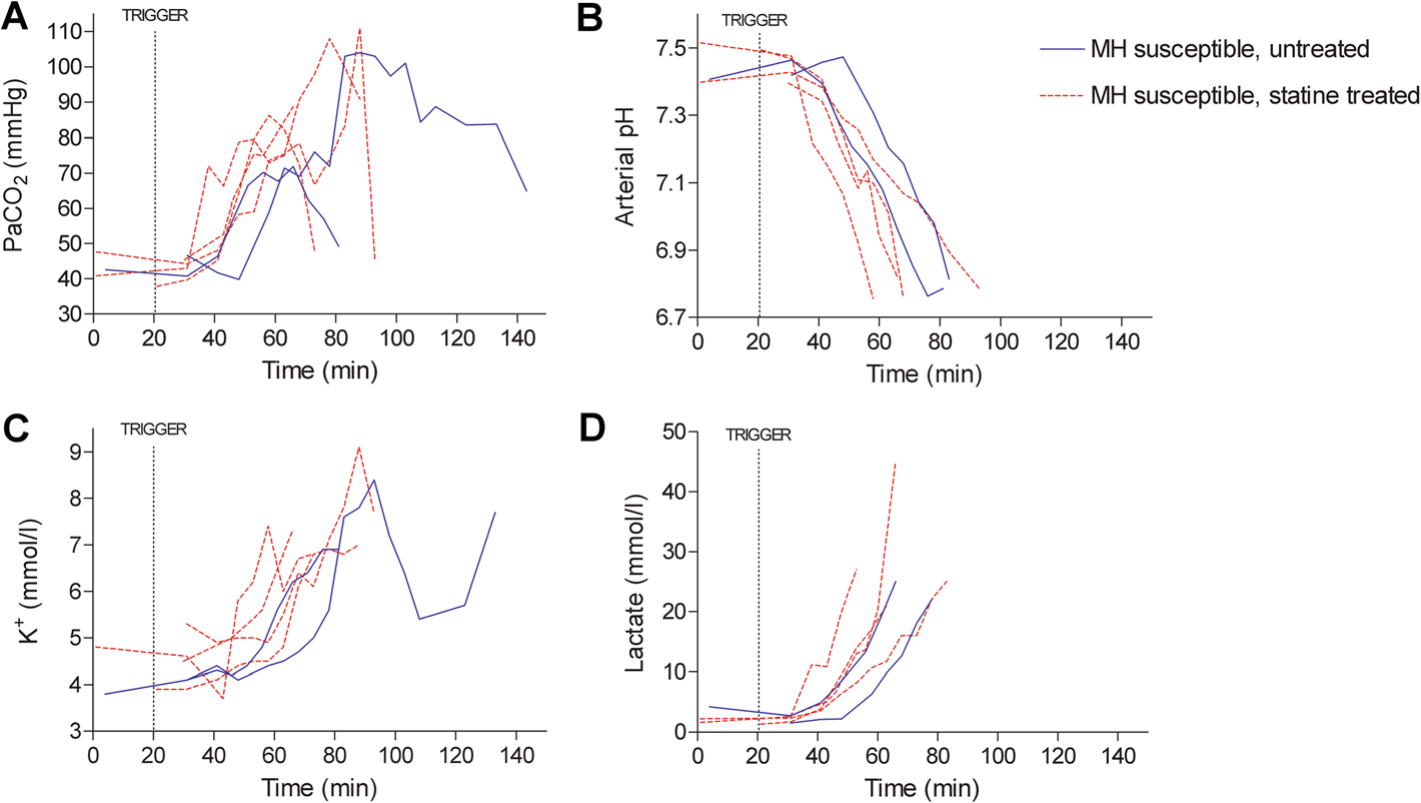
Laboratory investigations in arterial blood during sevoflurane-induced MH in susceptible swine treated with simvastatin (*N* = 4, discontinuous red line) and untreated susceptible swine (*N* = 2, blue line). The trigger line marks start of sevoflurane at inspired concentrations of 2.2%

MHS pigs tolerated the treatment with simvastatin without visible signs of toxicity. The raw data showing the progression of monitored cardiorespiratory, hemodynamic, thermal and metabolic measurements during sevoflurane anesthesia in each animal are given in Figures 2 and 3. MHN animals did not consistently reach the threshold for any variable. The time needed to reach the pre-defined thresholds for end-tidal pCO_2_ and MAP was on average shorter in statin-treated animals, and cardiac arrest occurred earlier (Table 3). Pulmonary artery temperatures presented variable departure values but were raised by minute 45 in statin treated animals, when untreated animals were still at basal temperature. Predefined thresholds for the monitored blood variables (PaCO_2_, pH, K+ and lactate concentrations) were also met sooner in statin-treated than in untreated MHS pigs (Table 3). These variables remained unchanged in MHN animals.

### Masseter spasm and foreleg rigor

**Fig. 4:**
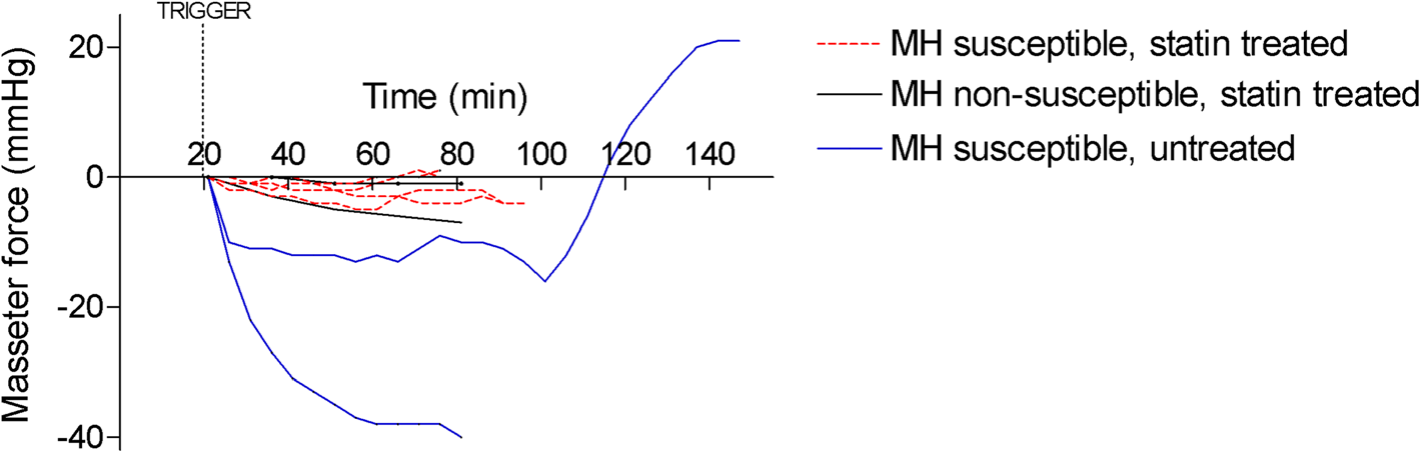
Masseter muscle force evolution after sevoflurane MH triggering in susceptible swine treated with simvastatin (*N* = 4, discontinuous red line), in MH susceptible swine with no statin treatment (*N* = 2, blue line) and in untreated MHN swine (*N* = 2, black line)

A prominent masseter relaxation was recorded upon sevoflurane administration in untreated MHS pigs, whereas untreated MHN animals showed only minor decreases in jaw pressure (Fig. 4). Afterwards, one of the untreated MHS pigs developed masseter spasm, while the other suffered cardiovascular collapse. The surviving animal also exhibited visible foreleg rigor, as is known in this animal model [19]. None of the statin treated MHS animals exhibited initial masseter relaxation, subsequent masseter spasm or visible limb rigor.

## Discussion

The disclosure of latent myopathies and MHS by statins, as well as evidence of statin myotoxicity in small animals has raised concerns that this drug class might adversely affect the outcomes of diagnostic MH susceptibility testing in vitro or the course of MH in vivo [16, 20]. We investigated these questions in genetically susceptible pigs, a well-documented model where both susceptibility and MH progression can be studied under settings similar to those used in humans. In addition, lipid metabolism in pigs resembles that of humans more closely than other species [21]. However, studies in big animals are logistically demanding, and a thorough study covering different statins, dosages and duration treatments would need considerable investment. Our aim was therefore to design a small prospective case study using a short-term treatment with a widely prescribed statin to capture the most salient features representing an average statin user.

In the context of susceptibility detection, no previous study in the literature has directly explored whether potential dysfunction of skeletal muscle induced by statin intake could impair the diagnostic efficacy of the IVCT. Metterlein et al. [12] detected an enhanced response of porcine MHS vs. MHN muscles to acute statin exposure in vitro, which does not inform about how prolonged statin therapy in vivo may affect IVCT outcomes. Our observations indicated that sensitivity to the agents used in human IVCT is not obscured by statin treatment in 3 muscles with a range of fiber type composition. Indeed, halothane-induced contractures were consistently enhanced, which could reflect a greater solubility of this lipophilic gas in the cholesterol-depleted muscle cell membranes of statin-treated animals, and eventually increase its concentration locally [22]. By contrast, caffeine-induced contractures were not altered by statin treatment, probably reflecting the different mechanisms triggered by this agent [23]. The sensitivity of MHS muscles to electrical field stimulation in vitro was not increased by statin treatment, except in diaphragm (Table 2), probably related to specific features of this muscle [24].

Simvastatin has been shown to promote the open conformation of RyR1 and RyR2 in lipid bilayers [11], raising the possibility that in the presence of this drug, decreased Ca^2+^-release thresholds might elicit contractures in normal skeletal muscle, compromising the specificity of the IVCT. Our experiments showed however, that muscles from simvastatin treated MHN pigs never responded with contractures to either halothane or caffeine. Increased muscle sensitivity to electrical stimulation was observed only in *rectus* muscles of treated MHN pigs (Table 2), which seems unrelated as contractures by trigger agents during IVCT were not elicited. Under standardized conditions similar to human testing, treatment with simvastatin did not compromise the discriminating power of the IVCT in swine.

In vitro viability of muscle bundles from statin treated (MHN or MHS) pigs, defined by twitch amplitude, was relatively lower than that from untreated animals, and may add technical challenge to the preparation of viable specimens in individuals under statins.

In vivo monitoring of sevoflurane-induced MH indicated faster development of hypercapnia, hemodynamic instability, lactic acidosis, hyperkalemia and asystole in statin treated MHS pigs. The differences are preliminary, given the small number of animals studied, but combining the rates at which these variables crossed pre-defined thresholds, together with earlier development of hyperthermia, suggests that deterioration may have been accelerated in the treated animals. Although one untreated MHS pig suffered premature cardiovascular collapse, presumably rushed by the unanticipated intervention (tracheotomy following failed intubation attempts), hypercapnia, tachycardia, hypotension, acidosis and hyperkalemia could still be recorded earlier in the experiment. The initial masseter relaxation recorded upon sevoflurane exposure, followed by subsequent spasm shown in susceptible Pietrain pigs were remarkably absent in statin treated pigs, which showed force dynamics that resembled those of MHN animals. Also, the muscle rigor that heralds MH in this model [19] was missing in treated animals. The findings are consistent with the muscle weakness and fatigability observed in individuals under statin medication [14] and suggests that impaired force development associated with statin intake may conceal rigor as a warning sign of upcoming MH. To understand the underlying mechanisms, it would be worth exploring this subject in additional models of statin myotoxicity, such as mice with genetic ablation of HMG-CoA (the enzyme targeted and inhibited by statins), which features a severe muscle phenotype [25]; or in newer models based on combined cholesterol-lowering therapies [26]. Statins have been proposed to disturb muscle function through impairment of energy metabolism driven by disturbed calcium homeostasis and mitochondrial dysfunction [3, 27, 28].

## Conclusions

To address concerns that statin-impaired muscle function could negatively affect outcomes of MH susceptibility testing, we show that statin treatment does not interfere with muscle contractures to halothane, which are rather enhanced. Both diagnostic sensitivity and specificity of the IVCT is unchanged by a short-term, moderate simvastatin intake.

However, the findings support previous views that statin therapy might complicate the clinical presentation of MH crises, if similar effects would extrapolate to humans. This is indicated by possibly accelerated metabolic deterioration and masked rigor in vivo. Clearly, adequately powered studies are needed to assess in detail the impact of cholesterol-lowering therapies on MH risk in susceptible individuals, and the results of this report should encourage further studies.

## Data Availability

The datasets used and/or analyzed during the current study are available from the corresponding author on reasonable request.

## Abbreviations

MH: Malignant hyperthermia
MHN: Malignant hyperthermia non-susceptible
MHS: Malignant hyperthermia susceptible
IVCT: In vitro contracture testing
RYR1: Ryanodine receptor type 1
CSA: Cross-sectional area
W: Bundle weight
L: Bundle length
